# The -160C>A Polymorphism in *e-Cadherin* Is Associated with the Risk of Nephrolithiasis

**DOI:** 10.1371/journal.pone.0073109

**Published:** 2013-09-02

**Authors:** Mingyue Tan, Shengqiang Xia, Qi Zhang, Jiang Zhu, Erdun Bao

**Affiliations:** 1 Department of Urology, Shanghai First People’s Hospital, Shanghai Jiao Tong University, Shanghai, China; 2 Department of Urology, Shanghai Tenth People’s Hospital, Tongji University, Shanghai, China; Nanjing Medical University, China

## Abstract

Nephrolithiasis is a common disorder worldwide. E-cadherin (*CDH1*) is involved in epithelial cell-cell interactions and plays important roles in the etiology of nephrolithiasis. We hypothesized that variants in the *CDH1* gene are associated with risk of nephrolithiasis. In a hospital-based case-control study of 127 nephrolithiasis patients and 152 controls frequency matched by age and sex, we genotyped the functional -160C>A (rs16260) polymorphism and assessed its associations with risk of nephrolithiasis in a Chinese population. We found that the CA/AA genotypes were associated with a significantly decreased risk of nephrolithiasis (OR = 0.53, 95%CI = 0.32-0.87), compared with the CC genotype, particularly among subgroups of BMI > 24 kg/m^2^ (OR = 0.38, 95%CI = 0.17-0.85), age ≤ 57 years (OR = 0.47, 95%CI = 0.24-0.93), and men (OR = 0.56, 95%CI = 0.29-0.99). Our results suggest that the *CDH1* polymorphism is involved in the etiology of nephrolithiasis and thus may be a marker for genetic susceptibility to nephrolithiasis.

## Introduction

Nephrolithiasis is a common nephrologic disorder, with a lifetime incidence of ~10% [1]. In China, the prevalence rate of nephrolithiasis is around 6% for men and 4% for women. Nephrolithiasis can cause severe acute back pain and sometimes leads to severe complications such as pyelonephritis or acute renal failure [2]. Nephrolithiasis is a complex disease resulting from an interaction between environmental and genetic factors. Although the etiology of nephrolithiasis is largely unknown, recent epidemiological investigations have showed that hypercalciuria, urinary tract infection, alkaline urine, westernized diet, and obesity are associated with nephrolithiasis [3–5]. However, only a part of the exposed individuals develop nephrolithiasis in their lifetime, suggesting that genetic susceptibility also play a role in nephrolithiasis.

E-cadherin (*CDH1*) plays a critical role in the establishment and maintenance of intercellular adhesion, cell polarity, and tissue architecture, which is expressed in almost all epithelial cells, including renal tubule cells [6,7]. Deregulation of *CDH1* in conjunction with renal cell injury, caused by oxalate crystals may contribute to the development of nephrolithiasis [8]. Polymorphisms within gene promoter regions can have profound effects on the transcriptional efficiency of genes. Several polymorphisms have been identified within the *CDH1* gene. Of these, a well known single nucleotide polymorphism (SNP) -160C>A (rs16260) has been identified in the promoter region, which has shown a 10~68% decreased level of transcriptional activity of the A allele compared to the C allele [9,10].

In spite of the advances in the knowledge of pathogenesis, little is know about the effect that genetic susceptibility has on the occurrence of nephrolithiasis. Because the *CDH1* gene is responsible for Ca^2+^-dependent cell-cell adhesion and plays an important for the establishment and maintenance of normal epithelial polarity and organization, we hypothesized that the *CDH1* -160C>A polymorphism is associated with risk of nephrolithiasis. To test this hypothesis, we investigated the correlation of *CDH1* gene polymorphism with risk of nephrolithiasis in a case-control study in a Chinese population.

## Materials and Methods

### Study subjects

Informed consent was obtained from all subjects and this study was approved by the Institute Review Board of Shanghai Jiaotong University. This was a hospital-based case-control study conducted at Shanghai First People’s Hospital of Shanghai Jiaotong University. A total of 127 nephrolithiasis cases and 152 controls were recruited, which were blood unrelated Han Chinese. All the patients were diagnosed on the basis of ultrasonographic and radiographic findings. Patients who showed symptoms of urinary tract infections, renal failure, chronic diarrhea, or cancer were excluded. The stone composition was analyzed by infrared spectroscopy. The controls were healthy volunteers with no history of familial urinary stone disease, and no history of renal calcification at health screening. All controls of appropriate age and sex for frequency matching with the cases were recruited and included if they gave their informed consent. After interview, about 5 mL venous blood sample was collected from each subject.

### Genotyping

Genomic DNA was isolated from leucocytes of venous blood by proteinase K digestion and phenol/chloroform extraction. The *CDH1* -160C>A polymorphism was determined by using the polymerase chain reaction-restriction fragment length polymorphism (PCR-RFLP) method. The PCR primers for the *CDH1* -160C>A polymorphism were 5’-TCCCAGGTCTTAGTGAGCCA-3’ (forward) and 5’-GGCCACAGCCAATCAGCA-3’ (reverse). The PCR reactions were performed in a total volume of 20µl containing 50ng genomic DNA, 1xTaq Buffer, 0.02mmol/L of MgCl_2_, 0.05mmol/L of dNTP mix, 10pmol/µl of each primer and 1U Taq DNA polymerase. After initial denaturation at 95^°^C for 5 min, the reaction was carried out at 95^°^C denaturation for 30 sec, 50^°^C annealing for 40 sec, and 72^°^C extension for 45 sec for a total of 34 cycles, and a final elongation at 72^°^C for 10 min. The amplified products were incubated with 5U of *Hph*I (New England Biolabs) restriction enzyme at 37^°^C overnight. The restriction fragments were then analyzed by electrophoresis in 3% agarose gel stained with 0.5% ethidium bromide and photographed under UV illumination. Genotype analysis was performed by two persons independently in a blind fashion. About 10% of the samples were randomly selected for repeated genotyping for confirmation, and the results were 100% concordant.

### Statistical analyses

Differences in the distributions of demographic characteristics and frequencies of genotypes of the *CDH1* -160C>A polymorphism between the nephrolithiasis cases and controls were evaluated by using the Student’s t-test (for continuous variables) or χ^2^-test (for categorical variables). The associations between the *CDH1* -160C>A genotypes and risk of nephrolithiasis were estimated by computing odds ratios (ORs) and their 95% confidence intervals (CIs) from unconditional logistic regression analysis with the adjustment for age and sex. Hardy-Weinberg equilibrium (HWE) was tested using a goodness-of-fit χ^2^-test. For the stratification by age, the age cutoff of 57 years was used in this study. *P* < 0.05 was considered statistically significant, and all statistical tests were two sided. All the statistical analyses were performed with the software SAS 9.1.3 (SAS Institute, Cary, NC, USA).

## Results

The frequency distributions of selected characteristics of the cases and controls are presented in Table 1. There were no differences between the cases and controls on age and sex (all *P* > 0.050). The age of the subjects ranged from 21 to 81 years. The mean age of the patients was 56.7 ± 10.2 years; the controls had a similar age distribution (57.3 ± 11.5 years). Men accounted for 65.4% of the patients and 63.2% of the controls. Furthermore, there was no significant difference in the body mass index (BMI) between the healthy subjects (23.8 ± 3.1 kg/m^2^) and nephrolithiasis patients (23.9 ± 3.0 kg/m^2^) (*P* = 0.831).

**Table 1:** Demographic and selected variables among nephrolithiasis cases and controls.

Variables	Cases (*n* = 127)			Controls (*n* = 152)		*P*
	*n*	%		*n*	%	
Age (years) (mean ± SD)	56.7 ± 10.2			57.3 ± 11.5		0.709
BMI (kg/m^2^) (mean ± SD)	23.9 ± 3.0			23.8 ± 3.1		0.831
Sex						
Male	83	65.4		96	63.2	0.709
Female	44	34.6		56	36.8	

As shown in Table 2, the frequencies of the CC, CA, and AA genotypes were 68.5, 26.8, and 4.7%, respectively, among the cases, and 53.9, 36.2, and 9.9%, respectively, among the controls (*P* = 0.034). The *CDH1* A allele frequency was 0.181 among the cases and 0.280 among the controls, and the difference was statistically significant (*P* = 0.006). The observed genotype frequencies among the controls were in agreement with the Hardy-Weinberg equilibrium (*P* = 0.209). Logistic regression analysis revealed that the CA genotype, but not the AA genotype, was associated with a significantly decreased risk of nephrolithiasis, compared with the CC genotype (OR = 0.57, 95%CI = 0.34-0.97 for CA versus CC; and OR = 0.37, 95%CI = 0.14-1.01 for AA versus CC). The A allele was associated with the decreased risk of nephrolithiasis in a dose–response manner (*P*_trend_ = 0.010). Furthermore, a significant decreased risk of nephrolithiasis was found in the combined genotypes CA/AA compared with the CC genotype (OR = 0.53, 95%CI = 0.32-0.87).

**Table 2:** Distribution of genotypes of *CDH1* -160C>A polymorphism and their associations with risk of nephrolithiasis.

Genotypes	Cases (*n* = 127)			Controls (*n* = 152)		*P*	OR (95% CI)	OR (95% CI)^a^
	*n*	%		*n*	%			
CC	87	68.5		82	53.9		1.00	1.00
CA	34	26.8		55	36.2	0.043	0.58 (0.35-0.98)	0.57 (0.34-0.97)
AA	6	4.7		15	9.9	0.055	0.38 (0.14-1.02)	0.37 (0.14-1.01)
CA/AA	40	31.5		70	46.1	0.013	0.54 (0.33-0.88)	0.53 (0.32-0.87)
A allele	0.181			0.280		0.006		
*P*_trend_						0.010		

^a^ Adjusted for age and sex.

We further evaluated the effect of *CDH1* -160C>A polymorphism on nephrolithiasis risk stratified by age, sex, and BMI (Table 3). We found that the decreased risk associated with the combined genotypes CA/AA were slightly more evident among young men with BMI > 24 kg/m^2^ (OR = 0.47, 95%CI = 0.24-0.93 for age ≤ 57 years, OR = 0.38, 95%CI = 0.17-0.85 for BMI > 24 kg/m^2^, and OR = 0.56, 95%CI = 0.29-0.99 among men). However, none significant gene-environment interaction was observed (data not shown).

**Table 3:** Stratified analyses on association between the *CDH1* -160C>A polymorphism and risk of nephrolithiasis.

Variables	CA/AA versus CC					Adjusted OR (95% CI)^a^
	CC			CA/AA		
	Cases	Controls		Cases	Controls	
	*n* (%)	*n* (%)		*n* (%)	*n* (%)	
Age (years)						
≤ 57	44 (65.7)	35 (46.7)	0.47 (0.24-0.93)	23 (34.3)	40 (53.3)	0.47 (0.24-0.93)
> 57	43 (71.7)	47 (61.0)	0.61 (0.29-1.26)	17 (28.3)	30 (39.0)	0.61 (0.29-1.26)
BMI (kg/m^2^)						
≤ 24	37 (58.7)	34 (45.3)	0.58 (0.29-1.14)	26 (41.3)	41 (54.7)	0.58 (0.29-1.14)
> 24	50 (78.1)	48 (62.3)	0.38 (0.17-0.85)	14 (21.9)	29 (37.7)	0.38 (0.17-0.85)
Sex						
Male	58 (70.0)	56 (58.3)	0.56 (0.29-0.99)	25 (30.0)	40 (41.7)	0.56 (0.29-0.99)
Female	29 (65.9)	26 (46.4)	0.47 (0.20-1.06)	15 (34.1)	30 (53.6)	0.47 (0.20-1.06)

^a^ Adjusted for age and sex.

## Discussion

In this hospital-based case-control study, we investigated the association of the *CDH1* -160C>A polymorphism and risk of nephrolithiasis in a Chinese population. We found that the CA/AA genotypes were associated with a significantly decreased risk of nephrolithiasis, and the association was more evident in young men with BMI > 24 kg/m^2^.

Hypercalciuria is one of the main risk factors for nephrolithiasis. Most patients with nephrolithiasis have calcium-containing stones [11]. Therefore, the physiological processes that influence calcium delivery to the kidney may influence stone formation. *CDH1*, an epithelial cellular junction protein expressed in renal tubule cells, contributes to the maintenance of epithelial development, organization, and cell integrity [8]. Li et al. (2000) reported that the -160C>A polymorphism has a significant effect on transcriptional activity of the promoter region; they demonstrated the in-vitro consequence of this polymorphism in decreasing gene transcription [9]. In particular, the A allele variant was demonstrated to reduce the transcriptional activity by 68%, indicating the possible role of this polymorphism as a novel genetic marker for nephrolithiasis. In the present study, we found that individuals who carried the combined CA/AA genotypes had a decreased risk of nephrolithiasis compared with those with the CC genotype. The possible explanation is that the A allele may result in the reduced urine calcium with the decreased formation of calcium oxalate crystals [12].

It has been reported that the some specific polymorphisms may be correlated with the increased risk of nephrolithiasis. For example, specific polymorphisms of the genes encoding vitamin D receptor (VDR) [13–16], vascular endothelial growth factor (VEGF) [17], androgen and estrogen receptors [18], calcium-sensing receptor (*CASR*) [19], matrix Gla protein (MGP) [20], and fibronectin (*FN 1*) [21] were suggested to be a risk factor for nephrolithiasis. For the *CDH1* polymorphism, Tsai et al. analyzed 148 patients with calcium oxalate stone and 103 healthy controls for the polymorphism in the *CDH1* 3’-UTR, they found that a significantly increased risk of nephrolithiasis in a dominant model [22]. However, in a case-control study composed of 143 patients with nephrolithiasis and 158 controls, Yilmaz et al. did not observe this polymorphism had an increased risk of nephrolithiasis among the Turkish population. In this study, we found that the -160C>A polymorphism was significantly associated with a decreased risk of nephrolithiasis in a Chinese population. The discrepancies between our current study and published studies might reflect differences in disease etiology and ethnic population [23]. Other factors such as selection bias, different matching criteria may also play a role.

Several limitations of this study should be addressed. Our study was hospital-base study design, we cannot rule the possible of selection bias of subjects that may have been associated with a particular genotype. However, we applied rigorous epidemiological design by limiting factors of selection subjects and used further statistical adjustment to minimize the potential biases. In addition, our sample size is moderate, and the statistical power of the study is limited, especially for subgroup and interaction analyses. Therefore, large population-based prospective studies with ethnically diverse populations are warranted to further elucidate the impact of *CDH1* polymorphism on nephrolithiasis susceptibility.
